# Hyperammonemia and Impaired Consciousness Caused by Non-Urease-Producing Actinotignum schaalii in Obstructive Urinary Tract Infection

**DOI:** 10.7759/cureus.76167

**Published:** 2024-12-21

**Authors:** Atsuo Nakamura, Masaharu Odo

**Affiliations:** 1 Emergency and Critical Care Medicine, Iizuka City Hospital, Iizuka, JPN

**Keywords:** alkaline urine, elderly patient, gram-positive rods, neurogenic bladder, non-urease-producing bacteria

## Abstract

Urinary tract infections (UTIs) caused by urease-producing bacteria are known to cause hyperammonemia; however, non-urease-producing bacteria can also cause it. This report describes a case of an 87-year-old woman who developed hyperammonemia and impaired consciousness resulting from a UTI caused by the non-urease-producing bacterium, *Actinotignum schaalii *(*A. schaalii*). On admission, the patient presented with urinary retention, hyperammonemia (281 μg/dL), and alkaline urine (pH 8.5). Gram staining of urine revealed the presence of gram-positive bacilli with coryneform morphology, which was suggestive of *A. schaalii *or *Corynebacterium*, with some being urease-producing bacteria. After bladder decompression through catheterization, the patient's level of consciousness improved within 30 minutes, and the ammonia level normalized. The patient's condition stabilized after ceftriaxone treatment, and she was discharged after nine days. This case demonstrated that non-producing bacteria can cause hyperammonemia and may acquire urease activity within an infected environment. In cases of hyperammonemia associated with obstructive urinary tract disorders, it is necessary to consider both urease-producing and non-urease-producing bacteria. Further, upon identification of a gram-positive bacillus, it is important to select an appropriate antimicrobial agent.

## Introduction

Hyperammonemia is a critical cause of impaired consciousness and is often associated with urinary tract infections (UTIs) caused by urease-producing bacteria [1-5]. However, non-urease-producing bacteria may also be causative agents [6-9]. We report a rare case of impaired consciousness due to hyperammonemia triggered by an obstructive UTI caused by the non-urease-producing bacterium, *Actinotignum schaalii* (*A. schaalii*).

## Case presentation

An 87-year-old woman who had undergone surgery for rectal cancer was discharged from the hospital three weeks prior to admission. She had a history of dementia and an overactive bladder, for which she was taking solifenacine succinate 5 mg and mirabegron 50 mg.

**Figure 1:**
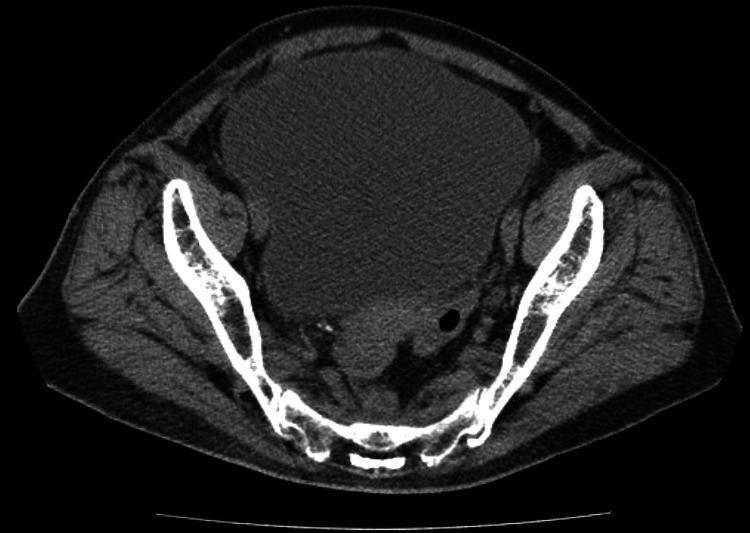
Abdominal CT on admission Abdominal CT revealed severe bladder distention

On admission, her vital signs were as follows: Glasgow Coma Scale (GCS) score, 8 (E1V2M5); blood pressure, 144/98 mmHg; heart rate, 110 beats per minute; respiratory rate, 18 breaths per minute; body temperature, 37.8 °C; and oxygen saturation (SpO₂), 93% on room air. Abdominal CT revealed severe bladder distention secondary to urinary retention (Figure 1). Laboratory tests revealed high blood ammonia levels (281 μg/dL) and a urine pH of 8.5, indicating alkaline urine (Table 1). Gram staining of the urine revealed gram-positive bacilli with coryneform morphology. Head CT revealed no brain edema or abnormalities.

**Table 1:** Laboratory data of patient on admission PTINR: Prothrombin time-international normalized ratio; APTT: Activated partial thromboplastin time

Parameters	Patient's values on admission	Normal values
Complete blood count		
White blood cell	9,400 /μL	3,300-8,600
Red blood cell	446 ×10^4^/μL	386-492
Hemoglobin	11.7 g/dL	11.6-14.8
Hematocrit	36.5%	35.1-44.4
Platelet	17.7×10^4^/μL	15.8-34.8
Arterial blood gas analysis (room air)		
pH	7.42	7.36-7.46
PO_2_	68 mmHg	85-105
PCO_2_	34.6 mmHg	35-45
HCO_3_^-^	22.2 mmol/L	24.2-29.8
Base excess	-2 mmol/L	-2.4-2.3
Biochemistry		
Total protein	6.8 g/dL	6.6-8.1
Albumin	3.5 g/dL	4.1-5.1
Total bilirubin	0.5 mg/dL	0.4-1.5
Aspartate transaminase	11 IU/L	13-30
Alanine transaminase	9 IU/L	7-23
Alkaline phosphatase	102 IU/L	38-113
Lactate dehydrogenase	153 IU/L	124-222
Blood urea nitrogen	25 mg/dL	8-20
Creatinine	0.8 mg/dL	0.46-0.79
Na	145 mEq/L	138-145
K	3.9 mEq/L	3.6-4.8
Cl	111 mEq/L	101-108
Glucose	177 mg/dL	73-109
C-reactive protein	0.39 mg/dL	0.00-0.14
Lactate	1.7 mmol/L	0.26-1.39
Ammonia	281 μg/dL	30-86
Coagulation system		
PTINR	0.91	0.87-1.09
APTT	24.6 sec	25-35
Fibrinogen	533 mg/dL	150-400
D-dimer	5.4 μg/ml	< 1.0
Urine analysis		
pH	8.5	-
Protein	1+	-
Occult blood	－	-
Ketones	－	-
Bacterium	3+	
White blood cells	30 /HPF	

A urethral catheter was inserted, and 1,500 mL of cloudy urine was drained. Her GCS score improved to 14 (E3V5M6) after 30 minutes, and her blood ammonia level decreased to 68 μg/dL after 6 hours. After 24 hours, her GCS score was 15, and her blood ammonia level was 56 μg/dL, with no subsequent increase. She received ceftriaxone 2 g for the UTI, and the urethral catheter was left in place given the diagnosis of a neurogenic bladder. The initial urine culture detected *A. schaalii*, but no bacteria were detected in subsequent urine cultures. The patient was discharged after nine days.

## Discussion

The causative bacterium in this case, *A. schaalii*, is a facultative anaerobic gram-positive bacillus exhibiting coryneform morphology [10]. It is a commensal organism commonly found in the urogenital area. Among 172 cases of human infections, 121 (70%) were found to involve UTIs. Although it generally has low virulence, *A. schaalii* can occasionally cause invasive infections, including bacteremia and endocarditis [10]. It grows poorly under standard culture conditions, requiring anaerobic conditions or 5% CO₂ environments for cultivation. Identification using matrix-assisted laser desorption/ionization time-of-flight mass spectrometry is considered useful [10].

**Figure 2:**
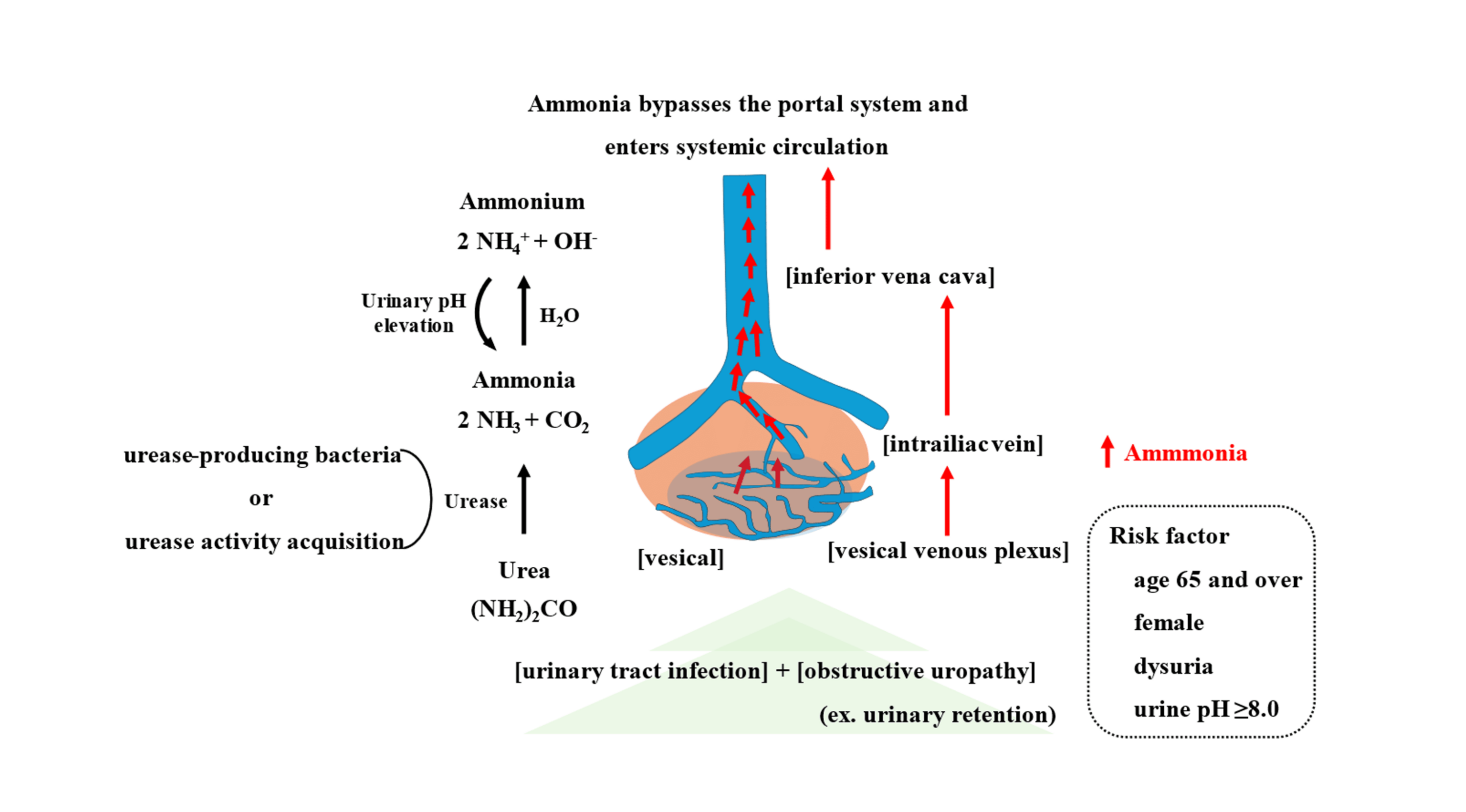
Pathogenesis of hyperammonemia due to UTI This is an illustration of the pathophysiology of a UTI that triggers hyperammonemia. This is an original image created by the authors. UTI: Urinary tract infection

Hyperammonemia caused by UTIs often involve women aged ≥65 years with underlying conditions causing urinary dysfunction, with nearly all cases being associated with obstructive uropathy [1,2,5,7-9]. A characteristic feature is alkaline urine, with a pH >8.0 [3-5,8,9] (Figure 2).

A study on the urease activity of urine-isolated bacteria found that >90% of positive strains were *Proteus mirabilis, Proteus vulgaris, Morganella morganii, Klebsiella pneumoniae*, and *Klebsiella oxytoca* [11]. Strains with <50% positivity included *Citrobacter freundii, Enterobacter cloacae, Pseudomonas aeruginosa, Staphylococcus epidermidis, Staphylococcus aureus*, and *Streptococcus faecalis*, whereas *Escherichia coli *showed no urease activity. Other bacteria with urease activity include *Corynebacterium urealyticum* [2]. *A. schaalii *lacks urease activity [10]. Causative agents of UTIs leading to hyperammonemia caused by non-urease-producing bacteria have been reported, including *Corynebacterium striatum*, *Escherichia colIi*, Alpha-hemolytic Streptococcus, *Staphylococcus epidermidis*, and *Streptococcus agalactiae *[7-9]. However, the underlying mechanisms remain unclear [7-9].

There are significant variations in urease activity among strains within the same species [11]. For example, *Staphylococcus epidermidis* strains range from strongly positive to negative. This suggests that urease activity is not a definitive factor for bacterial identification, and strains within the same species may exhibit varying urea-decomposing abilities [11]. In hyperammonemia due to UTIs, mechanisms other than urea decomposition by urease are unlikely. Therefore, urease activity may differ between laboratory culture conditions and individual infection environments, indicating that bacteria considered non-urease-producing may have acquired urease-producing abilities in the infection environment [9].

In the present case, two factors contributed to complete urinary retention leading to obstructive uropathy. First, urinary dysfunction was caused by Hartmann's procedure for rectal cancer. The patient had an abscessed enlarged lymph node on the right side of the rectal tumor, and partial damage to the pelvic plexus may have occurred during tumor dissection. During hospitalization, urination records showed repeated small amounts of in-diaper urination, and a CT scan at one month postoperatively revealed bladder distension. This suggests that she had been experiencing overflow incontinence due to incomplete urinary retention and that her dementia may have led to poor awareness of residual urine or urinary retention.

Second, the resumption of solifenacine succinate and mirabegron prescribed for an overactive bladder postoperatively contributed to complete urinary retention. Solifenacine succinate is an anticholinergic agent that suppresses bladder contraction. Mirabegron acts on β-adrenergic receptors, increasing bladder capacity, and post-marketing surveillance has reported urinary retention in 0.48% of patients with an overactive bladder [4,12]. During the perioperative period, with risks of bladder and rectal dysfunction, careful evaluation of urinary function and cautious resumption of medications are important.

Regarding treatment, though there was severe disturbance of consciousness, a characteristic feature is that bladder decompression by catheterization reduces ammonia levels and improves consciousness [3]. For infections caused by *A.* *schaalii*, beta-lactam antibiotics, which have low minimum inhibitory concentrations, are the first choice [11]. An important differential diagnosis includes infections caused by gram-positive bacilli of the genus* Corynebacterium*, for which macrolide or glycopeptide antibiotics are selected. At treatment initiation, antibiotic selection should also consider the coverage of *Corynebacterium* species.

## Conclusions

We describe a rare case of a UTI caused by the non-urease-producing bacterium, *A. schaalii*, which led to hyperammonemia and impaired consciousness. It is important to consider that non-urease-producing bacteria can be causative agents. When gram-positive bacilli are identified by urine gram staining, appropriate antibiotic selection for both *Corynebacterium* species and* A. schaalii *is important.
